# Mitochondrial translocation and interaction of cofilin and Drp1 are required for erucin-induced mitochondrial fission and apoptosis

**DOI:** 10.18632/oncotarget.2795

**Published:** 2014-12-16

**Authors:** Guobing Li, Jing Zhou, Amit Budhraja, Xiaoye Hu, Yibiao Chen, Qi Cheng, Lei Liu, Ting Zhou, Ping Li, Ehu Liu, Ning Gao

**Affiliations:** ^1^ College of Pharmacy, 3rd Military Medical University, Chongqing 400038, China; ^2^ State Key Laboratory of Natural Medicines, China Pharmaceutical University, Nanjing 210009, China; ^3^ Cell & Molecular Biology, St. Jude Children's Research Hospital, Memphis TN 38105, USA

**Keywords:** Cofilin, Drp1, mitochondrial fission, erucin, apoptosis

## Abstract

Cofilin is a member of the actin-depolymerizing factor (ADF) family protein, which plays an essential role in regulation of the mitochondrial apoptosis. It remains unclear how cofilin regulates the mitochondrial apoptosis. Here, we report for the first time that natural compound 4-methylthiobutyl isothiocyanate (erucin) found in consumable cruciferous vegetables induces mitochondrial fission and apoptosis in human breast cancer cells through the mitochondrial translocation of cofilin. Importantly, cofilin regulates erucin-induced mitochondrial fission by interacting with dynamin-related protein (Drp1). Knockdown of cofilin or Drp1 markedly reduced erucin-mediated mitochondrial translocation and interaction of cofilin and Drp1, mitochondrial fission, and apoptosis. Only dephosphorylated cofilin (Ser 3) and Drp1 (Ser 637) are translocated to the mitochondria. Cofilin S3E and Drp1 S637D mutants, which mimick the phosphorylated forms, suppressed mitochondrial translocation, fission, and apoptosis. Moreover, both dephosphorylation and mitochondrial translocation of cofilin and Drp1 are dependent on ROCK1 activation. *In vivo* findings confirmed that erucin-mediated inhibition of tumor growth in a breast cancer cell xenograft mouse model is associated with the mitochondrial translocation of cofilin and Drp1, fission and apoptosis. Our study reveals a novel role of cofilin in regulation of mitochondrial fission and suggests erucin as a potential drug for treatment of breast cancer.

## INTRODUCTION

Mitochondria are double membrane-bound organelles found in most eukaryotic cells, where they play essential and diverse roles in cellular physiology, including growth, division, energy metabolism, and apoptosis [1–3]. Mitochondrial morphology is regulated by the balance of two continuous antagonistic processes: fusion and fission [4]. Under physiological conditions, mitochondria are elongated and filamentous, but they undergo extensive fragmentation during apoptosis [5]. Apoptotic fission is associated with remodeling of the cristae, which is characterized by the opening of their tubular junction. This process results in the complete release of proapoptotic factors, such as cytochrome c, which is required in the cytosol for the activation of downstream effector caspases [6]. A number of evidence revealed that dynamin-related protein 1 (Drp1) participates in mitochondrial fission. Drp1 is a GTPase that causes scission of the mitochondrial outer membrane, resulting in fission of mitochondrial tubules into fragments [7, 8]. Drp1 translocates from the cytosol to the mitochondria and mediates mitochondrial fission prior to caspase activation and apoptosis [9]. Drp1 is responsible for cytochrome c release and caspase activation [10]. However, the mechanism by which Drp1 is recruited to mitochondria remains unresolved.

Cofilin is a member of the ADF/cofilin family of small actin-binding proteins found in all eukaryotic cells, and it regulates actin dynamics by increasing the rate of actin depolymerization and facilitating actin filament turnover [11]. Cofilin plays an essential role in regulation of the mitochondrial apoptosis [12]. Recent findings indicate that after induction of apoptosis, cofilin translocates from the cytosol to the mitochondria prior to the release of cytochrome c [12, 13]. Knockdown of cofilin results in the inhibition of both cytochrome c release and of apoptosis [12]. In addition, recent evidence has implicated cofilin in regulation of mitochondrial dynamics and functions [13]. Cofilin functions as a biosensor that integrates cytoskeletal regulation with mitochondrial function. The results of another study indicated that under certain cellular conditions cofilin might directly or indirectly associate with mitochondria and modulate mitochondrial functions [14]. However, the mechanism by which cofilin affects mitochondrial dynamics and functions, and is responsible for mitochondrial apoptosis remains unknown.

Rho-associated coiled coil-containing protein kinase 1 (ROCK1) belongs to a family of serine/threonine kinases that are activated by Rho GTPases or caspase-3 via cleavage the C-terminal auto-inhibitory domain away from the kinase active site [15, 16]. Recent studies have shown that ROCK1 plays an important role in regulation of apoptosis in various cell types and animal disease models [16–18]. It has been shown that ROCK1 plays a critical role in mitochondrial fission, which results in recruitment of dynamin-related protein-1 (Drp1) to the mitochondria [19]. ROCK1 has also been shown to play a critical role in dephosphorylation and mitochondrial translocation of cofilin [17]. However, the precise mechanism by which ROCK1 regulates the phosphorylation status of Drp1 and cofilin during mitochondrial fission and apoptosis remains unclear.

The natural compound 4-methylthiobutyl isothiocyanate (erucin) is found in consumable cruciferous vegetables and possesses chemopreventive and chemotherapeutic activities [20]. Increasing evidence suggests that erucin exerts its antiproliferative effects by inducing cell cycle arrest and apoptosis in various cancer cell lines *in vitro* [21, 22] and in tumor xenograft models *in vivo* [23]. The results of recent studies suggest that a mitochondrion-dependent pathway may play an important role in erucin-mediated apoptosis [24]. However, the molecular mechanisms by which erucin regulates the mitochondrial apoptosis pathway in human breast cancer cells has not yet been explored. Here, we report for the first time that erucin potently induced mitochondrial fission and apoptosis through mitochondrial translocation and interaction of cofilin and Drp1. Importantly, Rho-associated coiled coil-containing protein kinase 1 (ROCK1) was found to play an important role in regulating the dephosphorylation of cofilin and Drp1. Our *in vivo* findings indicated that the erucin-mediated inhibitory effects on tumor growth in a MDA-MB-231 xenograft mouse model was also associated with dephosphorylation and mitochondrial translocation of cofilin and Drp1, mitochondrial fission, and apoptosis. These findings provide a novel mechanistic basis for the application of erucin in the treatment of breast cancer.

## RESULTS

### Erucin induces apoptosis and mitochondrial fission in human breast cancer cells

**Figure 1:**
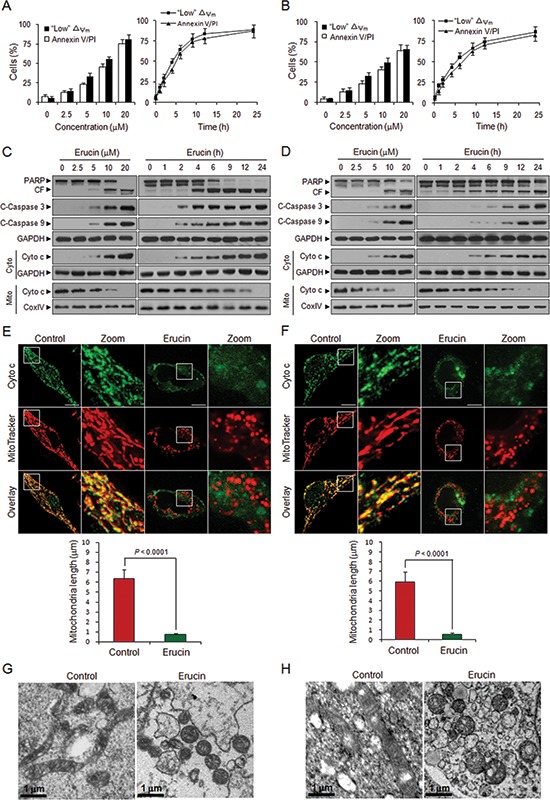
Erucin induces apoptosis and mitochondrial fission in human breast cancer cells **(A, B)** MDA-MB-231 (A) and MCF-7 (B) cells were treated with various concentrations of erucin for 9 h or 20 μM erucin for different time intervals as indicated. Apoptosis and loss of mitochondrial membrane potential (△Ψm) were determined by flow cytometry. **(C, D)** Whole cell lysates, mitochondrial (Mito) and cytosolic (Cyto) fractions from MDA-MB-231 (C) and MCF-7 (D) cells were prepared and subjected to immunoblotting using antibodies against PARP, cleaved-caspase 3 (C-Caspase 3), cleaved-caspase 9 (C-Caspase 9), cytochrome c (Cyto c), GADPH and Cox IV. **(E, F)** MDA-MB-231 (E) and MCF-7 (F) cells were treated with 20 μM erucin for 6 h, double-stained with Mitotracker Red CMXRos and cytochrome c (Alexa Fluor 488, green). Fluorescence images were collected by confocal microscopy. Scale bar represents 10 μm. Quantifications of mitochondrial length were performed as described in Methods. **(G, H)** MDA-MB-231 (G) and MCF-7 (H) mitochondrial morphology was evaluated by electron microscopy. Scale bar represents 1 μm.

First, we examined the effects of erucin on apoptosis and mitochondrial injury in human breast cancer MDA-MB-231 and MCF-7 cells. Flow cytometry analysis revealed that exposure of MDA-MB-231 and MCF-7 cells to erucin resulted in a significant increase in mitochondrial injury (loss of △Ψm) and apoptosis in dose- and time-dependent manners (Fig. 1A and 1B). Consistent with these findings, the same erucin concentrations and exposure intervals caused cleavage and activation of caspase 9 and caspase 3 and degradation of PARP. These events were also accompanied by significant increases in the release of cytochrome c from the mitochondria into the cytosol (Fig. 1C and 1D). Immunofluorescence assay also revealed that cytochrome c was release from mitochondria to cytosol after erucin treatment (Fig. 1E and 1F).

Mitochondrial fission is related to the initiation of apoptosis [4, 12, 25], and therefore, we examined the effects of erucin on mitochondrial fission in both MDA-MB-231 and MCF-7 cells. Mitochondria were labeled by staining with the mitochondrion-selective probe Mitotracker Red CMXRos. Exposure of cells to erucin resulted in significant decreases in the average length of mitochondria (Fig. 1E and 1F). The electron microscopic studies revealed the increased mitochondrial fragmentation, as evidenced by a significant increase in small, punctate mitochondria in erucin-treated cells compared with control cells, which exhibited elongated filamentous mitochondria (Fig. 1G and 1H). Taken together, these findings suggest that erucin induced mitochondrial fission, leading to the release of cytochrome c from mitochondria and cell death in human breast cancer cells.

### Erucin induces translocation of cofilin and Drp1 from the cytosol to mitochondria

**Figure 2:**
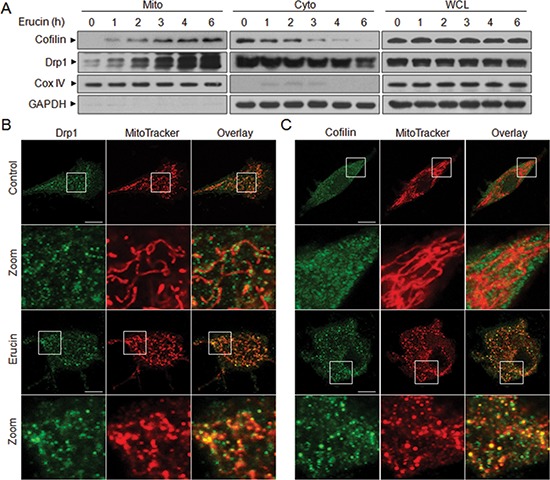
Erucin induces mitochondrial translocation of cofilin and Drp1 **(A)** MDA-MB-231 cells were treated with 20 μM erucin, whole cell lysates (WCL), mitochondrial (Mito), and cytosolic (Cyto) fractions were prepared and subjected to Western blot analysis. **(B, C)** Cells were treated with 20 μM erucin for 6 h, stained with Mitotracker Red CMXRos and Drp1 (Alexa Fluor 647, green) or cofilin (Alexa Fluor 488, green). Fluorescence images were collected by confocal microscopy. Scale bar represents 10 μm.

Recent evidence revealed that cofilin and Drp1 play critical roles in regulation of mitochondrial function by translocating from the cytosol to mitochondria [14, 17, 26–28]. We next investigated whether mitochondrial translocation of cofilin and Drp1 is necessary for erucin to induce mitochondrial fission. Treatment of cells with erucin significantly increased the levels of cofilin and Drp1 in mitochondria and decreased cofilin and Drp1 levels in the cytosol in a time-dependent manner (Fig. 2A). Immunofluorescence microscopy was used to further detect the sub-cellular localization of cofilin and Drp1 before and after erucin treatment. When cells were treated with erucin, cofilin and Drp1 signals were localized at the mitochondria (Fig. 2B and 2C). Mitochondrial fission was also observed following erucin treatment. These findings suggest that translocation of cofilin and Drp1 from the cytosol to the mitochondria is required for erucin-mediated mitochondrial fission.

### Erucin induces the interaction and colocalization of cofilin and Drp1 at the outer mitochondrial membrane

**Figure 3:**
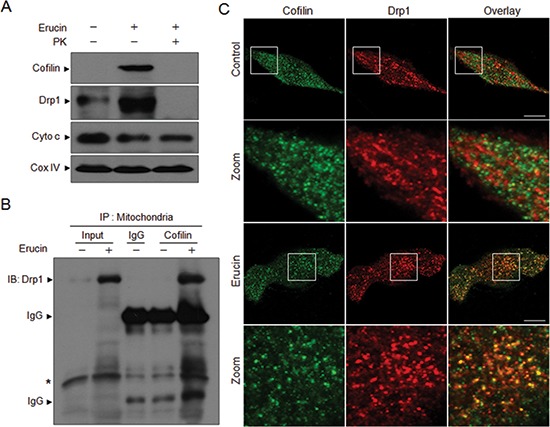
Erucin induces the interaction and colocalization of cofilin and Drp1 at the outer mitochondrial membrane **(A)** MDA-MB-231 cells were treated with 20 μM erucin for 6 h, mitochondria were purified and incubated with 10 ng/ml proteinase K on ice for 10 minutes, then centrifuged at 17,000g for 15 minutes. After washing, pellets were lysed in 2% SDS buffer and determined by immunoblotting. **(B)** Mitochondrial fractions were prepared and subjected to immunoprecipitation using anti-cofilin antibody, and the associated Drp1 were determined using immunoblotting. IgG represents the heavy (approximately 57 kDa) or the light (approximately 25 kDa) chain of cofilin antibody. Asterisk represents nonspecific band. **(C)** Cells were treated with 20 μM erucin for 6 h, stained with cofilin (Alexa Fluor 488, green) and Drp1 (Alexa Fluor 647, red) and evaluated by confocal microscopy. Scale bar represents 10 μm.

To determine whether cofilin and Drp1 were localized at the outer mitochondrial membrane, mitochondrial fractions from MDA-MB-231 cells were digested with proteinase K as described previously [12], cofilin and Drp1 proteins were analyzed by Western blot analysis. Both mitochondrial cofilin and Drp1 induced by erucin were digested completely by proteinase K, whereas Cyto c and CoxIV, which are internal mitochondrial markers are not digested by the protease treatment (Fig. 3A). These findings suggest that both cofilin and Drp1 are localized at the outer mitochondrial membrane after erucin treatment.

Because both cofilin and Drp1 participate in mitochondrial fission induced by erucin, we questioned whether cofilin could interact with Drp1 in response to erucin treatment in MDA-MB-231cells. Surprisingly, immunoprecipitation assay indicated that cofilin and Drp1 coimmunoprecipitated when cells were treated with erucin (Fig. 3B). Furthermore, immunofluorescence microscopic studies revealed a significant colocalization of cofilin and Drp1 in cells treated with erucin (Fig. 3C). Taken together, these findings indicate that the interaction and colocalization of cofilin and Drp1 at the outer mitochondrial membrane were essential for erucin-induced mitochondrial fission.

### Knockdown of cofilin decreases erucin-induced mitochondrial fission and apoptosis

**Figure 4:**
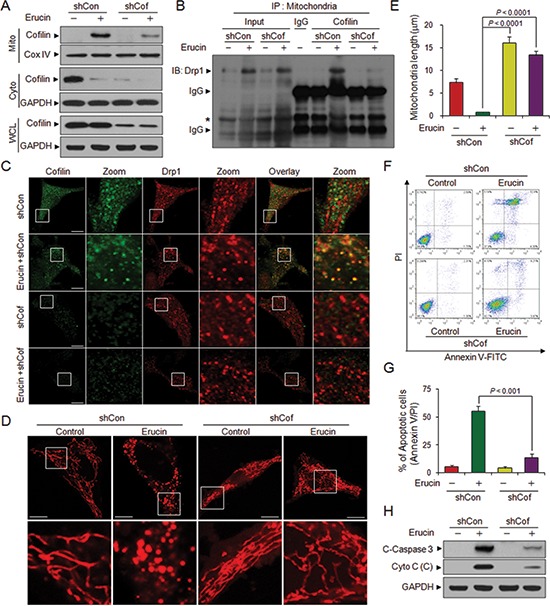
Cofilin knockdown decreases erucin-induced mitochondrial fission and apoptosis MDA-MB-231 cells were infected with lentivirus containing constructs that encoded scrambled control shRNA (shCon) or human cofilin-specific shRNA (shCof). Stable cell lines were treated without or with 20 μM erucin for 6 h. **(A)** Whole cell lysates (WCL), mitochondrial (Mito), and cytosolic (Cyto) fractions were prepared and subjected to immunoblotting. **(B)** Mitochondrial fractions were prepared and subjected to immunoprecipitation to determine the interaction of cofilin and Drp1. Asterisk represents nonspecific band. **(C)** Cells were immunostained with cofilin (Alexa Fluor 488, green) and Drp1 (Alexa Fluor 647, red). **(D, E)** Cells were stained with Mitotracker Red CMXRos and observed by confocal microscope. Scale bar represents 10 μm. Mitochondrial length was measured as described in Methods. **(F, G)** Apoptosis was measured by flow cytometry. **(H)** C-Caspase 3 in whole cell lysates and Cyto c in cytosolic fractions (C) were determined by immunoblotting.

To further address the potential role of cofilin in erucin-mediated mitochondrial fission, a lentivirus shRNA approach was used to stably knockdown cofilin expression. Knockdown of cofilin markedly reduced erucin-mediated cofilin translocation from the cytosol to mitochondria (Fig. 4A). Immunoprecipitation of the mitochondrial fraction revealed that knockdown of cofilin markedly reduced the interaction between cofilin and Drp1 mediated by erucin (Fig. 4B). Knockdown of cofilin also reduced the colocalization between cofilin and Drp1 induced by erucin (Fig. 4C). Knockdown of cofilin significantly abrogated erucin-mediated mitochondrial fission and resulted in mitochondrial elongation (Fig. 4D and 4E). Moreover, depletion of cofilin significantly abrogated erucin-mediated cytochrome c release, caspase 3 activation and apoptosis (Fig. 4F–4H).

### Knockdown of Drp1 decreases erucin-induced mitochondrial fission and apoptosis

**Figure 5:**
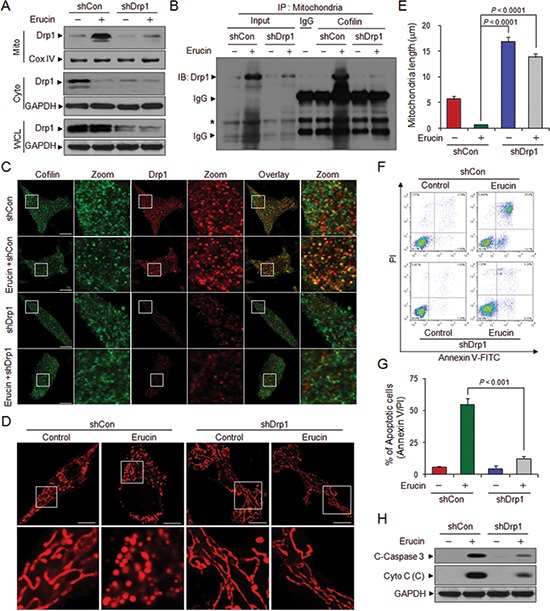
Drp1 knockdown decreases erucin-mediated mitochondrial fission and apoptosis MDA-MB-231 cells were infected with lentivirus containing constructs that encoded scrambled control shRNA (shCon) or human Drp1-specific shRNA (shDrp1). Stable cell lines were treated without or with 20 μM erucin for 6 h. **(A)** Whole cell lysates (WCL), mitochondrial (Mito) and cytosolic (Cyto) fractions were determined by immunoblotting. **(B)** Mitochondrial fractions were prepared and subjected to immunoprecipitation to determine the interaction of cofilin and Drp1. **(C–E)** Cells were immunostained with cofilin (Alexa Fluor 488, green) and Drp1 (Alexa Fluor 647, red) or Mitotracker Red CMXRos. Fluorescence images were collected by confocal microscopy. Scale bar represents 10 μm. Quantifications of mitochondrial length were performed as described in Methods. **(F, G)** Apoptosis was measured by flow cytometry. **(H)** C-Caspase 3 in whole cell lysates and Cyto c in cytosolic fractions (C) were determined by immunoblotting.

The translocation of Drp1 to mitochondria is a key event in mitochondrial fission [9, 26]. We next tested whether specific suppression of Drp1 with lentivirus shRNA affected erucin-induced mitochondrial fission and apoptosis. Infecting cells with Drp1 shRNA lentivirus significantly reduced Drp1 expression and erucin-mediated Drp1 translocation from the cytosol to the mitochondria (Fig. 5A). Drp1 knockdown also markedly reduced the interaction and colocalization between cofilin and Drp1 mediated by erucin compared to that in control shRNA cells (Fig. 5B and 5C). Knockdown of Drp1 inhibited erucin-mediated mitochondrial fission and resulted in mitochondrial elongation (Fig. 5D and 5E). Moreover, Drp1 suppression by shRNA significantly abrogated erucin-mediated cytochrome c release, caspase 3 activation and apoptosis (Fig. 5F–5H).

### Dephosphorylation of cofilin (Ser 3) and Drp1 (Ser 637) is required for erucin-mediated mitochondrial fission and apoptosis

**Figure 6:**
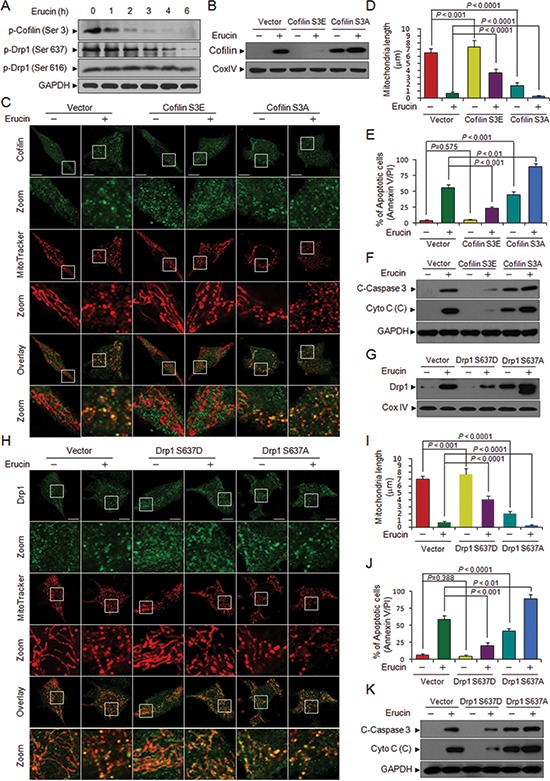
Dephosphorylation of cofilin (Ser 3) and Drp1 (Ser 637) is required for erucin-induced mitochondrial fission and apoptosis **(A)** MDA-MB-231 cells were treated with 20 μM erucin, whole cell lysates were determined by immunoblotting. **(B)** MDA-MB-231 cells were transfected with control empty vectors or human cofilin dephosphorylated (active, S3A) mutant plasmids or pseudophosphorylated (inactive, S3E) mutant plasmids for 48 h. After treatment with 20 μM erucin for 6 h, mitochondrial fractions were determined by immunoblotting. **(C, D)** Cells were double-stained with Mitotracker Red CMXRos and cofilin (Alexa Fluor 488, green). Fluorescence images were collected by confocal microscopy. Scale bar represents 10 μm. Mitochondrial length was measured as described. **(E)** Apoptosis was measured by flow cytometry. **(F)** C-Caspase 3 in whole cell lysates and Cyto c in cytosolic fractions (C) were determined by immunoblotting. **(G)** MDA-MB-231 cells were transfected with control empty vectors or human Drp1 dephosphorylated (active, S637A) mutant plasmids or pseudophosphorylated (inactive, S637D) mutant plasmids for 48 h. After treatment with 20 μM erucin for 6 h, mitochondrial fractions were determined by immunoblotting. **(H, I)** Confocal microscope images of transfected cells labeled with MitoTracker Red CMXRos and Drp1 (Alexa Fluor 647, green). Scale bar represents 10 μm. Mitochondrial length was measured as described. **(J)** Apoptosis was measured by flow cytometry. **(K)** C-Caspase 3 in whole cell lysates and Cyto c in cytosolic fractions (C) were determined by immunoblotting.

Recent studies have indicated that only dephosphorylated cofilin is translocated to mitochondria during the initiation of apoptosis [12]. Next, we investigated whether erucin could affect the phosphorylation status of cofilin. Treating cells with erucin resulted in the dephosphorylation of cofilin (Ser 3) in the total cellular extract in a time-dependent manner (Fig. 6A). To determine whether the phosphorylation status of cofilin could influence its ability to translocate to mitochondria and induce mitochondrial fission and apoptosis, two cofilin mutants mimicking either the dephosphorylated or phosphorylated forms were generated by changing Ser 3 to alanine (active; S3A) or glutamic acid (inactive; S3E) as described previously [29]. Overexpression of cofilin S3A enhanced, whereas cofilin S3E abolished, the mitochondrial translocation/localization of cofilin and mitochondrial fission mediated by erucin (Fig. 6B–6D). Furthermore, cofilin S3A significantly enhanced, whereas cofilin S3E reduced, erucin-mediated cytochrome c release, caspase 3 activation and apoptosis (Fig. 6E and 6F). Thus, our data indicated that the dephosphorylation of cofilin (Ser 3) mediated by erucin is required for cofilin translocation to mitochondria and increased mitochondrial fission and apoptosis.

Recent findings have indicated that dephosphorylation of Drp1 at Ser 637 causes Drp1 translocation to mitochondria and increases mitochondrial fission [26, 30]. Phosphorylation of Drp1 at Ser 616 by Cdk1/Cyclin B promotes mitochondrial fission during mitosis [31]. We then examined the effect of erucin on phosphorylation of Drp1 at Ser 637 and Ser 616. Exposure of cells to erucin resulted in significantly decrease in the levels of phospho-Drp1 at Ser 637, but had no effect on phosphorylation of Drp1 at Ser 616 (Fig. 6A). To further examine the role of Drp1 Ser 637 dephosphorylation in erucin-induced mitochondrial fission and apoptosis, we generated a mutant of Drp1 Ser 637D (S637D) to mimic the constitutively phosphorylated form or a mutant of Drp1 Ser 637A (S637A) to mimic the dephosphorylated form. Overexpression of Drp1 S637A significantly increased, whereas Drp1 S637D decreased, the mitochondrial translocation/localization of Drp1 and mitochondrial fission mediated by erucin (Fig. 6G–6I). Moreover, Drp1 S637A significantly increased, whereas Drp1 S637D reduced, erucin-mediated cytochrome c release, caspase 3 activation and apoptosis (Fig. 6J and 6K). These findings indicated that dephosphorylation of Drp1 Ser 637 is required for erucin-induced mitochondrial translocation of Drp1, mitochondrial fission and apoptosis.

### ROCK1 is involved in erucin-induced dephosphorylation and mitochondrial translocation of cofilin and Drp1, mitochondrial fission, and apoptosis

**Figure 7:**
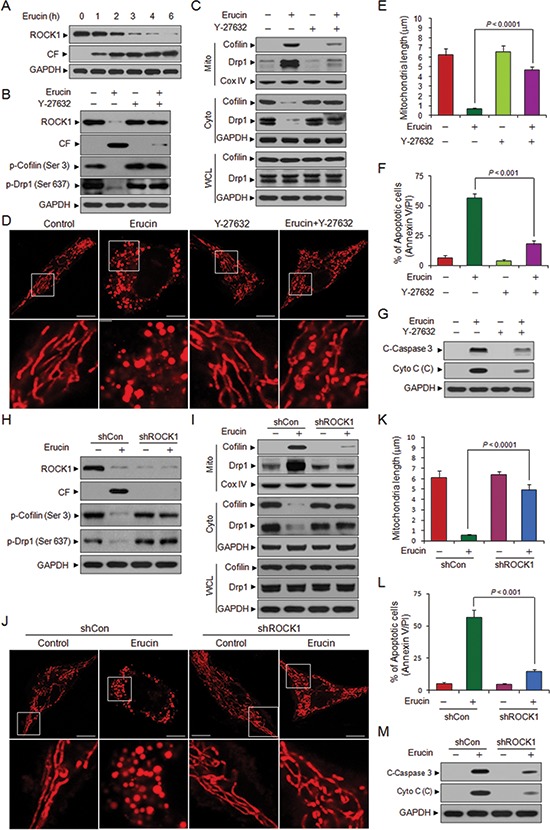
Inhibition of ROCK1 activation or knockdown of ROCK1 attenuate erucin-induced cofilin and Drp1 dependent mitochondrial fission and apoptosis **(A)** MDA-MB-231 cells were treated with 20 μM erucin, whole cell lysates were determined by immunoblotting. **(B, C)** MDA-MB-231 cells were pretreated with 20 μM Y-27632 for 2 h, followed by treatment with 20 μM erucin for 6 h. Whole cell lysates (WCL), mitochondrial (Mito) and cytosolic (Cyto) fractions were determined by immunoblotting. CF represents cleavage fragment. **(D, E)** Cells were stained with Mitotracker Red CMXRos and observed by confocal microscope. Scale bar represents 10 μm. Mitochondrial length was measured as described. **(F)** Apoptosis was measured by flow cytometry. **(G)** Whole cell lysates and cytosolic fractions were determined by immunoblotting. **(H, I)** MDA-MB-231 cells were infected with lentivirus containing constructs that encoded scrambled control shRNA (shCon) or human ROCK1-specific shRNA (shROCK1). Stable cell lines were treated without or with 20 μM erucin for 6 h. Whole cell lysates (WCL), mitochondrial (Mito) and cytosolic (Cyto) fractions were probed by immunoblotting. **(J, K)** Cells were stained with Mitotracker Red CMXRos and observed by confocal microscope. Mitochondrial length was measured as described. Scale bar represents 10 μm. **(L)** Apoptosis was measured by flow cytometry. **(M)** Whole cell lysates and cytosolic fractions were determined by immunoblotting.

It has been shown that cofilin can be dephosphorylated by the phosphatases PP1 and PP2A, which are regulated by the ROCK1 signaling pathway [17, 32–35], and phosphorylated by LIM kinase, which is also regulated by ROCK1 [36–38]. ROCK1 is involved in the regulation of Drp1 phosphorylation status and promote Drp1 translocation to mitochondria, leading to mitochondrial fission [19]. We next examined whether erucin affects the expression of ROCK1, PP1, PP2A, LIMK1, LIMK2 and phospho-LIMK1(Thr508)/LIMK2(Thr505). Treating cells with erucin resulted in a marked decrease in the levels of ROCK1 and increased cleavage of ROCK1 in a time-dependent manner (Fig. 7A). Exposure of cells to erucin also resulted in marked increase in levels of PP1 and PP2A in time-dependent manners. However, the levels of LIMK1, LIMK2 and phospho-LIMK1(Thr508)/LIMK2(Thr505) were not altered by erucin treatment (Supplementary Fig. S1).

To further assess the functional significance of ROCK1 activation in regulating the phosphorylation status and mitochondrial translocation of cofilin and Drp1, a ROCK1 inhibitor Y-27632 was employed. Pretreating cells with Y-27632 significantly decreased erucin-mediated ROCK1 cleavage/activation and dephosphorylation of cofilin (Ser 3) and Drp1 (Ser 637) (Fig. 7B). Pretreatment with Y-27632 also markedly decreased erucin-mediated translocation of cofilin and Drp1 from the cytosol to mitochondria (Fig. 7C). Y-27632 also significantly decreased erucin-induced mitochondrial fission (Fig. 7D and 7E). Moreover, Y-27632 significantly attenuated erucin-mediated cytochrome c release, caspase 3 activation and apoptosis (Fig. 7F and 7G).

To further confirm these results, a lentivirus shRNA approach was used to stably knockdown ROCK1 expression. Knockdown of ROCK1 significantly blocked erucin-mediated dephosphorylation of cofilin (Ser 3) and Drp1 (Ser 637) (Fig. 7H), Knockdown of ROCK1 also blocked erucin-mediated translocation of cofilin and Drp1 from the cytosol to mitochondria (Fig. 7I). ROCK1 suppression by shRNA significantly inhibited erucin-mediated mitochondrial fission (Fig. 7J and 7K). Furthermore, depletion of ROCK1 also significantly decreased erucin-mediated cytochrome c release, caspase 3 activation and apoptosis (Fig. 7L and 7M). Taken together, these findings indicated that activation of ROCK1 played a critical role in erucin-mediated dephosphorylation and mitochondrial translocation of cofilin and Drp1, mitochondrial fission, and apoptosis.

### Erucin inhibits tumor growth in a MDA-MB-231 xenograft mouse model

**Figure 8:**
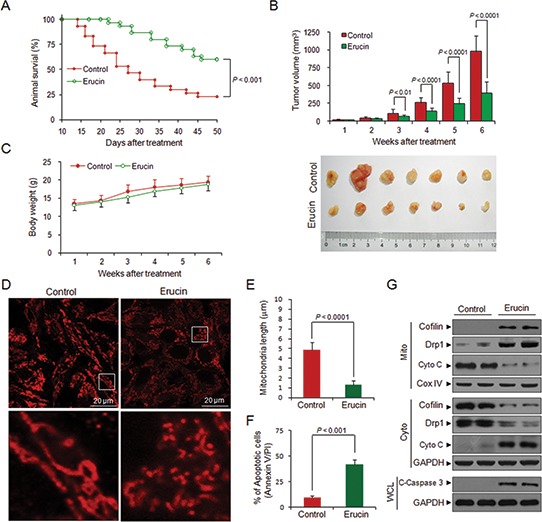
Erucin inhibits tumor growth of MDA-MB-231 xenograft model **(A)** The comparison of animal survival between erucin-treated group and vehicle-treated group. *p*-values were calculated using Kaplan–Meier method. **(B)** Average tumor volume in vehicle control mice and mice treated with erucin. *p* < 0.01 or *p* < 0.0001 compared with vehicle control. **(C)** Body weight changes of mice during the 50 days of erucin treatment. **(D, E)** Primary cells were isolated from representative tumor tissues and cultured in DMEM medium and stained with Mitotracker Red CMXRos. Fluorescence images were collected by confocal microscopy. Mitochondrial length was measured as described. Scale bar represents 20 μm. **(F)** Apoptosis of primary cells was measured by flow cytometry. **(G)** Whole cell lysates (WCL), mitochondrial (Mito) and cytosolic (Cyto) fractions from representative tumor tissues in two vehicle-treated mice and two erucin-treated mice were prepared and subjected to Western blot analysis.

To determine whether our *in vitro* findings could be replicated *in vivo*, nude mice were inoculated subcutaneously with MDA-MB-231 cells followed by injections with vehicle or erucin (50 mg/kg, i.p.) for 50 days starting one week after tumor inoculation. Treatment with erucin resulted in a significant increase in survival compared with untreated controls (*P* < 0.001) (Fig. 8A). Treatment with erucin resulted in a significant suppression of tumor growth after 3 weeks of drug exposure (*P* < 0.01 or *P* < 0.0001 vs vehicle control) (Fig. 8B). However, there were no significant changes in body weight (Fig. 8C) or other signs of potential toxicity such as agitation, impaired movement and posture, indigestion or diarrhea.

To evaluate the effect of erucin on mitochondrial dynamics in the MDA-MB-231 xenograft *in vivo*, we used immunofluorescence microscopy to examine the morphology of mitochondria in primary cells isolated from the MDA-MB-231 xenograft in vehicle control or erucin-treated mice. Following treatment with erucin, mitochondria became short and fragmented, as evidenced by a significant decrease in the average length of mitochondria (Fig. 8D and 8E). Moreover, apoptosis and Western blot analyses showed that treatment with erucin significantly increased cytochrome c release, caspase 3 activation and apoptosis in primary cells isolated from the MDA-MB-231 xenograft (Fig. 8F and 8G).

Western blot analysis was used to further evaluate whether mitochondrial translocation of cofilin and Drp1 occurred during erucin-mediated mitochondrial fission and apoptosis in primary cells. Cofilin and Drp1 were found in mitochondrial fractions following treatment with erucin, concomitant with the disappearance of cofilin and Drp1 from cytosolic fractions (Fig. 8G). Thus, our data indicated that mitochondrial translocation of cofilin and Drp1 occurred during erucin-induced mitochondrial fission and apoptosis *in vivo*.

## DISCUSSION

The results of the present study demonstrated for the first time that erucin potently induced apoptosis by triggering mitochondrial fission and that this phenomenon was due primarily to the interaction and recruitment of cofilin and Drp1 to mitochondria. Cofilin is a member of the ADF/cofilin family, which regulates actin dynamics by increasing the rate of actin depolymerization [11]. Recent evidence has demonstrated that mitochondrial translocation of cofilin induces the release of cytochrome c and apoptosis in neutrophils and neuroblastoma and lymphoma cells [12, 39]. However, the mechanism by which cofilin affects mitochondrial injury remains elusive. Our data showed that erucin-mediated mitochondrial fission and apoptosis was accompanied by mitochondrial translocation of cofilin. Knockdown of cofilin markedly reduced erucin-mediated mitochondrial fission and apoptosis. Thus, these data suggest that mitochondrial translocation of cofilin is crucial for erucin-mediated mitochondrial fission and apoptosis.

The most interesting finding of the present study was that the interaction and colocalization of cofilin and Drp1 at mitochondria was involved in erucin-mediated mitochondrial fission during apoptosis. The dynamin-related protein Drp1 is essential for most types of mitochondrial fission [26, 40]. Drp1 is a cytosolic GTPase that has been proposed to couple GTP hydrolysis to membrane constriction and fission [41]. Recent evidence has revealed that Drp1 translocates to mitochondria and mediates mitochondrial fission [42]. Drp1 homo-oligomerizes and forms a ring around the mitochondrial tubule [43]. Drp1 complexes might generate mechanical force via conformational changes, leading to membrane constriction and fission [44]. The role of cofilin in erucin-mediated Drp1 recruitment and mitochondrial fission is strongly supported by several lines of evidence. First, both cofilin and Drp1 translocated from the cytosol to mitochondria during erucin-induced mitochondrial fission. Second, cofilin and Drp1 interacted and colocalized at the outer mitochondrial membrane during erucin-mediated mitochondrial fission. Third, knockdown of cofilin or Drp1 significantly blocked the interaction and colocalization between cofilin and Drp1, mitochondrial fission and apoptosis. To the best of our knowledge, this is the first report to demonstrate that the interaction and mitochondrial translocation of cofilin and Drp1 are required for erucin-mediated mitochondrial fission and apoptosis.

Here, we report that erucin-induced mitochondrial fission depends on the phosphorylation status of cofilin and Drp1. The regulation of cofilin involves the phosphorylation and dephosphorylation of its Ser 3 residue; cofilin is inhibited by phosphorylation [45] and translocates to mitochondria only in its dephosphorylated (active) form [12]. The dephosphorylated form of cofilin (S3A) promotes oxidant-mediated mitochondrial damage and apoptosis [39]. Consistent with these findings, our data indicate that only dephosphorylated cofilin translocates to mitochondria. Interestingly, we found that constitutively active cofilin S3A (dephosphorylated) enhanced, whereas the dominant-negative cofilin S3E (phosphorylated) blocked, the mitochondrial translocation of cofilin, mitochondrial fission and apoptosis induced by erucin. Thus, erucin-mediated dephosphorylation of cofilin at Ser 3 is required for the translocation of cofilin to mitochondria and increased mitochondrial fission and apoptosis.

The dynamin-related protein Drp1 is essential for most types of mitochondrial fission [4, 26]. The majority of the available evidence has revealed that regulation of Drp1 by post-translational modifications is important for Drp1 translocation to mitochondria [46, 47]. It has been reported that calcineurin-dependent dephosphorylation of Drp1 at Ser 637 regulates its translocation to mitochondria and subsequent mitochondrial fission [48]. The increased dephosphorylation of Drp1 at Ser 637 regulates its translocation to mitochondria and induces mitochondrial fission, which leads to an increased response to apoptotic stimuli [49–51]. However, another report demonstrated that phosphorylation of Drp1 Ser 600 (corresponding to Ser 637 in human) of mouse podocytes promotes Drp1 translocation to mitochondria and increases mitochondrial fragmentation [19]. Our findings demonstrate that dephosphorylation of Drp1 at Ser 637 regulates its translocation to mitochondria and induces mitochondrial fission in response to erucin based on the following evidence. First, erucin induced dephosphorylation of Drp1 at Ser 637 but not Ser 616. Second, overexpression of Drp1 mutant S637A (dephosphomimetic) increased, whereas Drp1 mutant S637D (phosphomimetic) reduced, the mitochondrial translocation of Drp1, fission and apoptosis. Thus, dephosphorylation of Drp1 at Ser 637 by erucin is required for the translocation of Drp1 to mitochondria and increased mitochondrial fission and apoptosis.

The present study demonstrated that activation of ROCK1 was essential for dephosphorylation of cofilin (Ser 3) and Drp1 (Ser 637), which promoted cofilin and Drp1 recruitment to mitochondria and mitochondrial fission. Recent studies have shown that ROCK1 plays an important role in regulation of apoptosis in various cell types [16, 17]. ROCK1 has diverse functional activities in different cell types [18, 37]. ROCK1 activation can regulate activation/dephosphorylation of cofilin by induction of PP1 and PP2A phosphatase activities [17, 32], or control phosphorylation of cofilin by LIM kinase [36–38]. Our data indicate that erucin-induced ROCK1 activation and subsequent activation/dephosphorylation of cofilin are mainly caused by increasing the PP1 and PP2A phosphatase activities. The LIM kinase seems not to be involved in this process. It has been shown that ROCK1 plays an important role in remodeling mitochondrial morphology by regulating the phosphorylation and mitochondria translocation of Drp1 in podocytes [19]. Our previous study showed that ROCK1 activation plays a critical role in regulating cofilin mitochondrial translocation and apoptosis in leukemia cells [17]. Our findings suggest that activation of ROCK1 plays a critical role in erucin-mediated dephosphorylation and mitochondrial translocation of cofilin and Drp1, mitochondrial fission and apoptosis, based on the following evidence: (i) Erucin induced activation of ROCK1 in a time-dependent manner; (ii) Inhibition of ROCK1 activity by Y-27632 or knockdown of ROCK1 by shRNA attenuated erucin-mediated cofilin and Drp1 dephosphorylation and mitochondrial translocation; (iii) Inhibition of ROCK1 activity by Y-27632 or knockdown of ROCK1 by shRNA significantly reduced erucin-induced mitochondrial fission and apoptosis.

In summary, the present findings demonstrate for the first time that erucin induces mitochondrial fission and apoptosis in breast cancer cells through mitochondrial translocation and interaction of cofilin and Drp1. Collectively, these observations suggest a hierarchy of events in erucin-induced apoptosis in which ROCK1 activation represents the primary event, leading to the dephosphorylation of cofilin (Ser 3) and Drp1 (Ser 637), which are translocated from the cytosol to the mitochondria, culminating in mitochondrial fission, cytochrome c release and apoptosis.

## MATERIALS AND METHODS

### Antibodies and chemicals

The following antibodies were used for Western blot analysis: Drp1 (5391, 1:1,000 working dilution), cleaved-caspase 3 (9661, 1:1,000), cleaved-caspase 9 (9505, 1:1,000 ), Cox IV (4850, 1:2,000), phospho-cofilin (Ser 3) (3313, 1:500), phospho-Drp1 (Ser 637) (4867, 1:500), phospho-Drp1 (Ser 616) (3455, 1:500), LIMK1 (3842, 1:2,000), LIMK2 (3845, 1:1,000) and phospho-LIMK1(Thr508)/LIMK2(Thr505) (3841, 1:500) were purchased from Cell Signaling Technology; GAPDH (sc-25778, 1:500), cytochrome c (sc-13156, 1:1,000), cofilin (sc-376476, 1: 2,000) and PP1 (sc-7482, 1:1,000) were purchased from Santa Cruz Biotechnology; PARP (ab32071, 1:1,000) and ROCK1 (ab45171, 1:1,000) were from Abcam; PP2A (610555, 1:5,000) was purchased from BD Biosciences. Erucin (sc-204741) and Y-27632 (sc-216067) were purchased from Santa Cruz Biotechnology.

### Cell culture

MDA-MB-231 and MCF-7 cells were purchased from the American Type Culture Collection, who authenticates cell lines with short tandem repeat profiling and monitoring cell morphology. Cells were maintained in Dulbecco's Modified Eagle Medium (DMEM) containing 10% FBS at 37°C in 5% CO_2_ and passaged for less than 6 months after receipt.

### Lentiviral gene transfer and gene silencing

The human cofilin1 shRNA (5′-CCGGAAGGTGTTCAATGACATGAAACTC GAGTTTCATGTCATTGAACACCTTTTTTTG-3′)and human Rock1 shRNA (5′-CCGGGCACCAGTTGTACCCGATTTACTCGAGT AAATCGGGTACAACTGGTGCTTTTTG-3′) were synthesized and subcloned into the pLKO.1 plasmid; Human DRP1 shRNA plasmid (sc-43732) and control shRNA plasmid (sc-108060) were purchased from Santa Cruz Biotechnology (Santa Cruz, CA, USA). Plasmids were co-transfected with lentiviral packaging vectors (pLP1, pLP2 and pLP/VSVG) into 293FT cells using Lipofectamine 3000 according to the manufacturer's instructions. The lentivirus-containing supernatant was harvested and was used to infect the MDA-MB-231 cells. Cells were subsequently grown under 5 μg/ml puromycin selection to establish stable cell lines.

### Site-directed mutagenesis and transfection

Dephosphorylated (active, S3A) and pseudophosphorylated (inactive, S3E) human cofilin constructs were a gift from Professor James Bamburg (Colorado State University, USA); dephosphorylated (active, S637A) and pseudophosphorylated (inactive, S637D) Drp1 mutants were generated using the QuickChange Site-Directed Mutagenesis Kit (Stratagene, CA, USA) with the following primers: S637A (FW: 5′-AGTTCCTGTTGCACGAAACTAGCTGCTCGGG AAC-3′; RV: 5′-GTTCCCGAGCAGCTAGTTTT CGTGCAACAGGAACT-3′), S637D (FW: 5′-CCAGTTCCTGTTGCACGAAAACTAGATGCTC GGGAACAGCGAGATTGTGAG-3′; RV: 5′-CTCA CAATCTCGCTGTTCCCGAGCATCTAGTTTTCGTG CAACAGGAACTGG-3′). The mutated Drp1 was confirmed by sequence analysis. Plasmids were transfected into MDA-MB-231 cells using Lipofectamine 3000 according to the manufacturer's instructions. After 48 h of transfection, the cells were exposed 20 μM erucin for 6 h and subsequently subjected to immunoblotting or immunofluorescence analysis.

### Apoptosis and mitochondrial membrane potential assay

Apoptotic cells were detected using the Annexin V-FITC/PI staining kit (PharMingen, San Diego, CA) as previously described [17]. The mitochondrial membrane potential (△Ψm) was monitored using 3,3′-dihexyloxacarbocyanine (DiOC_6_, Molecular Probes), briefly, after treatment, cells were incubated with 40 nM DiOC_6_ at 37°C for 15 minutes, washed twice with PBS and subsequently analyzed by flow cytometry (Becton-Dickinson, San Jose, CA) with an excitation wavelength of 488 nm and an emission wavelength of 530 nm to determine the percentage of cells exhibiting low levels of DiOC_6_ uptake.

### Mitochondrial and cytosolic fraction preparation

Mitochondrial and cytosolic fractions were obtained as described previously [17].

### Immunoprecipitation and western blot analysis

Cells were lysed in 1% NP-40 buffer (50 mM Tris (pH 7.4), 150 mM NaCl, 1% Nonidet P-40, 10% glycerol, 1 mM PMSF, 10 μg/ml aprotinin, 10 μg/ml leupeptin, 1 mM Na3VO4). Equal quantities of proteins were initially precleared by incubation with non-specific normal mouse IgG (Pierce Biotechnology) and protein A/G agarose beads (Pierce Biotechnology) for 1 h. After centrifugation, the supernatant was incubated with cofilin antibody overnight at 4°C. Immunoprecipitates were then collected using protein A/G agarose beads followed by several washes in lysis buffer. The samples were separated by SDS-PAGE, transferred to PVDF membranes, and processed for immunoblotting as previously described [17].

### Immunofluorescence

Cells were grown on 12-mm coverslips, stained with 500 nM Mitotracker Red CMXRos (M7512, Molecular Probes) for 30 min at 37°C and washed twice with PBS. The cells were then fixed with 4% formaldehyde for 15 min, permeabilized with 0.1% Triton X-100 for 10 min, and blocked with 1% BSA in PBS for 30 min. Immunostaining was performed using the antibodies including anti-cofilin (1:50, sc-376476, Santa Cruz Biotechnology), anti-Drp1 (1:50, 5391, Cell Signaling Technology) and anti-cytochrome c (1:50, sc-13156, Santa Cruz Biotechnology), followed by the appropriate secondary antibodies Alexa Fluor 488 goat anti-mouse (A11001, Molecular Probes) or Alexa Fluor 647 donkey anti-rabbit (A31573, Molecular Probes), for 1 h at room temperature. Images were collected using a Leica scanning confocal microscope (TCS SP2 AOB, Wetzlar, Germany). Mitochondrial length was measured as previously described [52]. Briefly, the Mitotracker-stained regions in cells with clearly resolved mitochondria were measured using the line tool in the Leica Application Suite (version: 2.4.1) software. For each group, approximately 100 mitochondria from at least five different cells were counted.

### Transmission electron microscopy

Cells were harvested by trypsinization and fixed with ice-cold 4% glutaraldehyde in PBS (pH 7.4) at 4°C overnight. Next, the cells were postfixed in 2% osmium tetroxide, dehydrated in a graded series of ethanol, and embedded in Epon 812 resin. Ultra-thin sections (0.05 μm) were obtained using glass knives and double-stained with uranyl acetate/lead citrate. Finally, the cells were observed using a Hitachi-7500 electron microscope (Hitachi Instrument, Tokyo, Japan).

### Xenograft assay

Animal studies were performed according to federal guidelines and approved by the Third Military Medical University Institutional Animal Care and Use Committee. Nude mice (5 weeks old) were obtained from Vital River Laboratories (Beijing, China). MDA-MB-231 cells (2 × 10^6^ cells/mouse) were suspended in serum-free DMEM and injected subcutaneously into the right flank of each mouse. Mice were randomized into two groups (*n* = 30 per group). One week after tumor inoculation, the mice received either erucin (50 mg/kg, i.p., five times per week) or an equal volume of vehicle. Signs of potential toxicity, such as agitation, impaired movement and posture, indigestion or diarrhea, and areas of redness or swelling were observed during the treatment. Tumor volume, body weight and survival time were measured at various time intervals throughout the study. Tumor volumes were calculated according to the formula (width^2^ × length)/2. Mice were sacrificed at the termination of the experiment, and tumor tissues from representative mice were lysed and subjected to Western blot analysis. Primary cells were isolated from tumor tissues, cultured in DMEM and then subjected to immunofluorescence and apoptosis analysis.

### Statistical analysis

All data values are represented as mean ± SD. Statistical analysis was performed using two-tailed Student's *t*-tests. Survival analysis in the xenograft experiment was performed using the Kaplan–Meier method, and significance was calculated using the log-rank test.

## SUPPLEMENTARY FIGURE


