# Supplementary material for: Mitochondrial translocation and interaction of cofilin and Drp1 are required for erucin-induced mitochondrial fission and apoptosis
Source: Oncotarget. 2014 Dec 16;6(3):1834–49. doi: 10.18632/oncotarget.2795 (PMC4359335; doi:10.18632/oncotarget.2795)
Supplement: Supplementary file 1 [file oncotarget-06-1834-s001.pdf]

SUPPLEMENTARY FIGURE

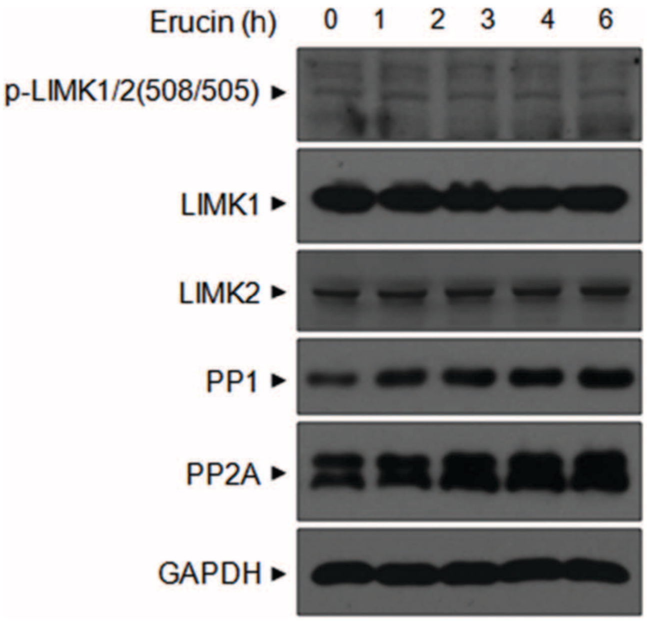

Supplementary Figure S1: The phosphatases PP1 and PP2A, but not LIM kinase, are involved in dephosphorylation of cofilin and Drp1 induced by erucin. MDA-MB-231 cells were treated with 20 µM erucin for 0, 1, 2, 3, 4 and 6 h. Total cellular extracts were prepared and subjected to Western blot assay using antibodies against PP1, PP2A, LIMK1, LIMK2, phospho-LIMK1(Thr508)/LIMK2(Thr505) and GAPDH.
